# Evaluation of the Effectiveness of Nordic Walking Training in Improving the Gait of Persons with Down Syndrome

**DOI:** 10.1155/2019/6353292

**Published:** 2019-04-15

**Authors:** Agnieszka Skiba, Jakub Marchewka, Amadeusz Skiba, Szymon Podsiadło, Iwona Sulowska, Wiesław Chwała, Anna Marchewka

**Affiliations:** ^1^Department of Clinical Rehabilitation, University of Physical Education in Cracow, al. Jana Pawła II 78, 31-571 Cracow, Poland; ^2^Department of Biomechanics, University of Physical Education in Cracow, al. Jana Pawła II 78, 31-571 Cracow, Poland

## Abstract

People with Down syndrome (DS) show dysfunction of gait, expressed by disturbed character of angular changes and values of the spatiotemporal parameters as compared to the physiological norm. It is known that exercises and various activities have positive effect on balance and gait, but there are only a few scientific proofs concerning above-mentioned in people with DS. Furthermore, the effect of Nordic Walking (NW) training on gait in people with DS is unexplored. We enrolled 22 subjects with DS, aged 25-40 years, with moderate intellectual disability. Participants were randomly divided into 2 groups: NW training group which underwent 10 weeks of training at a frequency of 3 times a week and control group with no specific intervention. Subjects were examined twice: 1 week before training and a week immediately after intervention. Gait was evaluated by the Vicon 250: a computerized system of three-dimensional analysis of motion, connected to 5 infrared video cameras. We conducted mixed-design ANOVA model to assess the effects of time and type of training on spatiotemporal parameters. We found significant favorable time by group interaction in the following parameters: step length in right leg: F(1,15) =14,47, p=0.002; left leg accordingly F(1,15) =5,15, p=0.038, cycle length in right leg: F(1,15) =14,48, p=0.002; left leg accordingly F(1,15) =15,09, p=0.001; and gait standardised speed F(1,15) =5,35, p=0.035. Statistically significant changes were observed in numerous kinematic parameters of ankle, knee, pelvis, and shoulder in NW group. Regular NW training has positive influence on selected spatiotemporal and kinematic parameters in people with Down Syndrome and may be an attractive and safe form of rehabilitation.

## 1. Introduction

Adult persons with Down Syndrome (DS) present impairment of the motor skills involving gait that differs from the physiological standard. Characteristic disadvantageous changes in the gait comprise spatiotemporal parameters, such as decreased gait speed, longer double support phase, reduced step length, and increased step width. Typical unfavourable changes also include reduced or absent alternating upper limbs movement [1, 2]. The ability to control the gait seems to reflect the previously acquired motor experiences; therefore applying diverse sensory and focused training may help to modify the motor responses and improve the gait of persons with DS [3, 4].

Engaging in regular physical activity by persons with DS may have a considerable effect on their lifespan and their physical, mental, and emotional health. Studies show positive effect of motor training on the physical activity of persons with an intellectual disability [5, 6]. Mendonca et al. [7] compared the effects of combined aerobic and resistance training in adult persons with DS and in the healthy adults. In their study, observed benefits of the physical activity involved an improved exercise capacity and gait economy. What is more, the magnitude of these changes was similar in both groups. It has also been proven that a higher level of physical activity in persons with DS significantly reduces the incidence of cardiovascular diseases and helps increase the independence of these persons [8, 9]. The available research results allow for an optimistic perspective on improving the gait and balance of persons with DS through physical activity. Nordic Walking (NW) seems to be an attractive and safe form of physical activity for persons with DS. Firstly, increasing the area of the base of support with poles improves the stability and balance. Another advantage of this type of training is its higher intensity: the training activates the muscles of the upper and lower body and, at the same time, prevents a strain on the motor system. Regular NW training increases the mobility of joints, improves physical fitness, and facilitates the correction of body posture. It has been proven that NW training is effective for evaluating physical fitness, gait, balance, and exercise tolerance, has a positive effect on the quality of life, and decreases the risk of falls among elderly persons [10–15]. Many experimental studies have shown that physical activity performed in the form of NW training has a positive effect on the health of patients without an intellectual disability. Reuter et al. [16] proved that NW training improved the gait speed and increased the distance covered in patients with Parkinson's Disease. In addition, Mannerkorpi et al. [17] proved that brisk NW was effective in improving the functional fitness of persons with fibromyalgia.

However, the available literature lacks reports on the effect of NW training on the gait of persons with DS. The following research hypothesis was set forth: NW training decreases disturbances of gait in people with DS and allows for values of the angular changes in the joints closer in values to the variables achieved by healthy people. The study assumed that NW training has positive influence on people with DS concerning spatiotemporal parameters of gait and angular changes in the joints. The aim of this study was to evaluate the effect of NW training on the gait of persons with DS and to compare the effectiveness of the NW training with a group of participants not undergoing NW training. Detailed aims included investigating the effect of the therapy on the spatiotemporal parameters of gait and on angular changes in the joints of persons with DS.

## 2. Materials and Methods

### 2.1. Ethical Approval

The study was approved by the Local Chamber of Physicians and Dentists Bioethics Committee in Cracow, approval no: 69/KBL/OIL/2016. All the procedures complied with the Helsinki Declaration.

### 2.2. Participants

We enrolled 22 adults (11 women and 11 men) with Down Syndrome with a moderate intellectual disability (IQ 36–51), aged between 25 and 40 years, and the mean age was 31,18 ± 4,1 years. Participants were recruited from the Occupational Therapy Workshops in Cracow. The study cohort was randomly divided into two subgroups: Group NW (n=11 persons), which underwent a 10-week Nordic Walking training programme, and Group C (n=11 persons), the control group, which did not undergo any training during the 10-week period. In Group NW, the mean age was 30.3 ± 2.8 years, and the mean body height was 1.56 ± 0.69 m; in Group C, the mean age was 32.0 ± 5.21 years while the mean body height was 1.52 ± 1.02 m. No statistically significant differences at the baseline were observed concerning spatiotemporal parameters between the experimental group (Group NW) and the control group (Group C).

### 2.3. Gait Analysis

Gait was analysed using the Vicon 250 Optoelectronic System for three-dimensional Motion Analysis, which can register the movements of the studied person with a frequency of 120 Hz. The device is composed of five cameras that receive infrared signals and a workstation connected to a computer, which collects and analyzes data with the WorkStation, BodyBuilder, and Polygon software. Gait is recorded through changes in the location of 39 passive markers, attached to anthropometric points of the body according to the Golem model (Oxford Metrics Ltd.) [18, 19]. The main stage of the assessment involved walking along an outlined section 20 metres long at a natural speed, without any stimulation during the movement. The qualified persons were assessed twice: in the week preceding the start of the programme (Before) and in the week directly following the programme (After). The study was conducted in the Biokinematics Laboratory of the Department of Biomechanics of the University of Physical Education in Cracow under the same conditions and at the same time of day. Gait was evaluated based on the analysis of changes in the spatiotemporal parameters and in the peak values of angles in the selected joints in the experimental group and the control group. These results are presented in comparison to the results obtained for the physiological gait (N). The reference data (N) was collected at the Biokinematics Laboratory of the Department of Biomechanics of the University of Physical Education in Cracow. This data includes the results of the evaluation of the gait in 12 healthy persons (6 women and 6 men, aged between 25 and 40 years). The groups were not differentiated according to gender, because, due to the mutual relations between the gait speed, length, and frequency of steps, persons with a similar somatic body build and similar values of the spatiotemporal parameters of gait were qualified into their respective groups, regardless of gender [18]. The results of the evaluation of angular changes are presented, according to the phases of a normalised gait cycle, in figures generated by the Polygon software. The mean values of the angular changes and the comparison between the right and the left side of the body of the studied persons are presented in comparison to the band representing two standard deviations from the mean value of the angle of the reference data. The figures present the mean angular changes in the sagittal plane in the ankle joint, knee joint, hip joint, and shoulder joint for the right and the left limbs. The figures also present the angular changes of the pelvis in three planes.

### 2.4. Therapeutic Procedure

The therapeutic procedure encompassed 10 weeks of 60-minute training sessions conducted three times a week. Each session was composed of a warm-up (10 min), main part (45 min), and a cool-down phase (5 min). The warm-up constituted the introductory part of the training session: it was performed in a standing position, and its aim was to prepare the body for an increased effort. The protocol also involved exercises requiring alternate work with the upper and the lower limbs, such as alternately raising the lower limbs to touch the opposite pole. The main part involved brisk NW. The cool-down phase comprised breathing and stretching exercises. The intensity of the brisk walking progressed during the course of the training sessions. The exercises were conducted by a physical therapist, who was a qualified NW instructor.

### 2.5. Statistical Analysis

We conducted mixed-design analysis of variance model to assess the effects of time, group, and time and type of training on spatiotemporal parameters. Our between-subjects factor consists of treatment (NW or controls), and our within-subjects factor was time; participants were evaluated at the baseline: before and after the intervention. Normality of the data was assessed by Shapiro Wilk test, homogeneity of variance by Levene's test, and sphericity by Mauchly's Test. For pairwise comparisons when applicable we used t-tests or Wilcoxon signed-rank test. Calculations were performed using Statistica 12.5 (StatSoft® Inc. USA). P<0.05 was considered statistically significant.

## 3. Results

We found significant interaction time and group effects in the following spatiotemporal parameters: step length, cycle length, speed, standardised (STD) step length, and standardised (STD) speed between the analysed cohorts over time as shown in Tables 1 and 2.

All other time, group and time by group effects concerning analysed spatiotemporal parameters were not significant.

**Figure 1:**
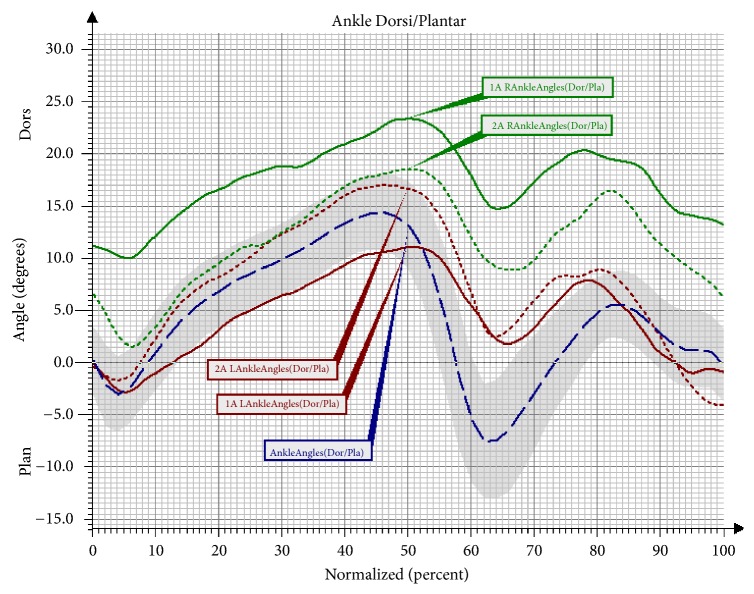
Angular changes in the ankle joint in the sagittal plane in Group NW before and after the training. Ankle Angles: mean angular changes in the reference group; 1A Ankle Angles: mean angular changes in the ankle joint before the therapy; 2A Ankle Angles: mean angular changes in the ankle joint after the therapy; Dor/Dors: dorsiflexion; Pla/Plan: plantar flexion; L: left; R: right.

We observed desynchronised movement of the right and the left ankle joint in the first assessment, compared to the angle changes in the group of healthy persons. It was noted that the angular changes in both joints were different in the first assessment, while, in the second assessment, the curves of the angular changes lay closer to one another (Figure 1). In addition, statistically significant changes were noted for the right lower limb in the Stance Phase (SP). The difference involved an excessive dorsiflexion in the phase, which should involve a motion towards the plantar flexion in the ankle joint. After the training, the peak angular value in the Loading Response phase approached the reference values, reaching the plantar flexion (Table 3).

**Figure 2:**
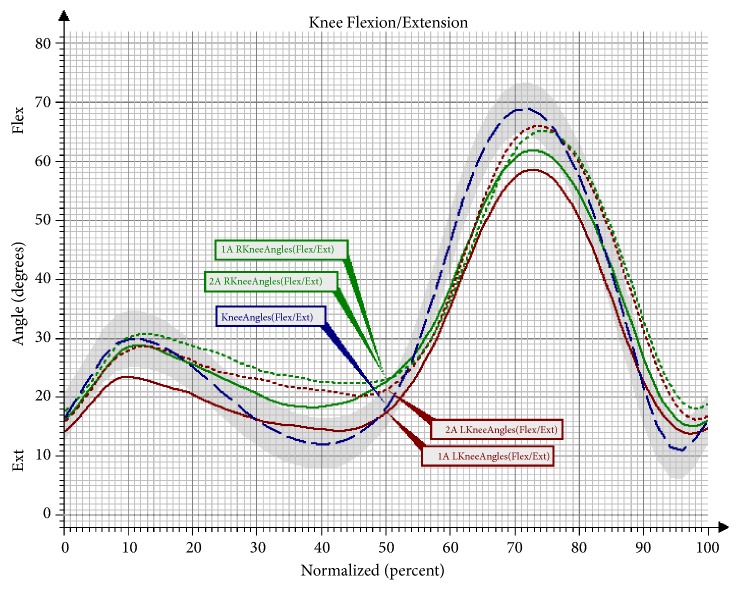
Angular changes in the knee joint in the sagittal plane in Group NW before and after the training. Knee Angles: mean angular changes in the reference group; 1A Knee Angles: mean angular changes in the knee joint before the therapy; 2A Knee Angles: mean angular changes in the knee joint after the therapy; Flex: flexion; Ext: extension; L: left; R: right.

The comparison of changes in the knee joint indicates an increase of the range of flexion movements in both limbs after the conducted therapy. In the second assessment, the mean peak values fell within the range determined by the model of physiological gait (Figure 2). A statistically significant change was observed for the left lower limb, in which there was an increase of the range of flexion in the knee joint in the Mid-Stance (MS) phase, the Terminal Stance (TS) phase, and the Initial Swing (ISw) phase (Table 4).

**Figure 3:**
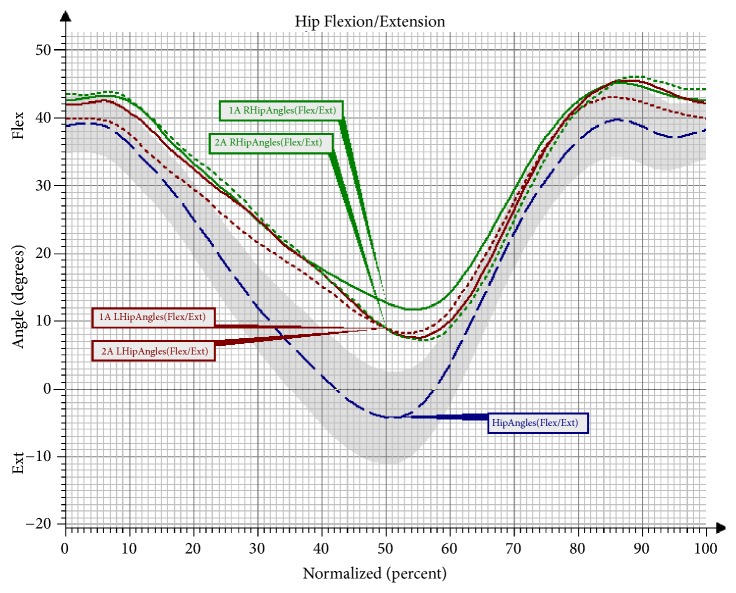
Angular changes in the hip joint in the sagittal plane in Group NW before and after the training. Hip Angles: mean angular changes in the reference group; 1A Hip Angles: mean angular changes in the hip joint before the therapy; 2A Hip Angles: mean angular changes in the hip joint after the therapy; Flex: flexion; Ext: extension; L: left; R: right.

For the hip joint, a delay of synchronisation of motion was observed in the study in both the right and the left hip joint in both assessments, when compared to the changes in the angle in the reference group of healthy persons (Figure 3). No significant differences were noted in any of the gait phases; however, there was a notable trend for increased motion towards extension in the Pre-Swing phase (PSw) for the right limb after the training (Table 5).

**Figure 4:**
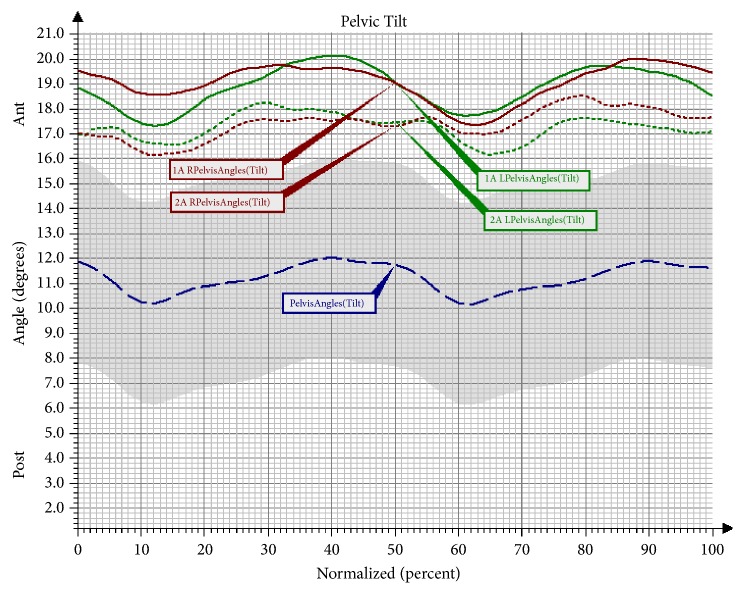
Angular changes in the movement of the pelvis in the sagittal plane in Group NW before and after the training. Pelvis Angles: mean angular changes in the reference group; 1A Pelvis Angles: mean angular changes in the pelvis before the therapy; 2A Pelvis Angles: mean angular changes in the pelvis after the therapy; Ant: anterior tilt; Post: posterior tilt; L: left; R: right.

In the study, a significant desynchronisation of the movement of the pelvis in the sagittal plane was noted compared to the angular changes in the group of healthy persons in both assessments, although the mean range of angular changes after the NW training approached the reference ranges (Figure 4). Significant differences were also noted in the movement of the pelvis in the sagittal plane in the MS phase for the right limb and in the ISw phase for the left limb (Table 6).

**Figure 5:**
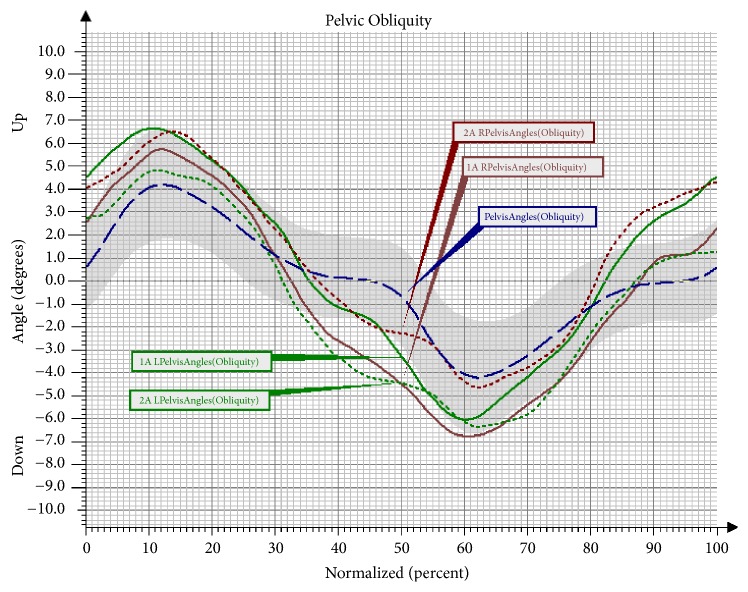
Angular changes in the movement of the pelvis in the frontal plane in Group NW before and after the training. Pelvis Angles: mean angular changes in the reference group; 1A Pelvis Angles: mean angular changes in the pelvis before the therapy; 2A Pelvis Angles: mean angular changes in the pelvis after the therapy; L: left; R: right.

For the changes in the movement of the pelvis in the frontal plane, there were delays observed in the study in the synchronisation of movement for the right and left side and a modest desynchronisation in the first assessment compared to the reference curves. After the NW training, both peak values of the angular deviations of the pelvis in the frontal plane were within the reference norms (Figure 5). Significant differences were also observed in the movement of the pelvis in the frontal plane at the maximal point of movement for the left limb and in the minimal point of movement for the right limb. The value of the amplitude displayed a downward trend in the second assessment (Table 7).

**Figure 6:**
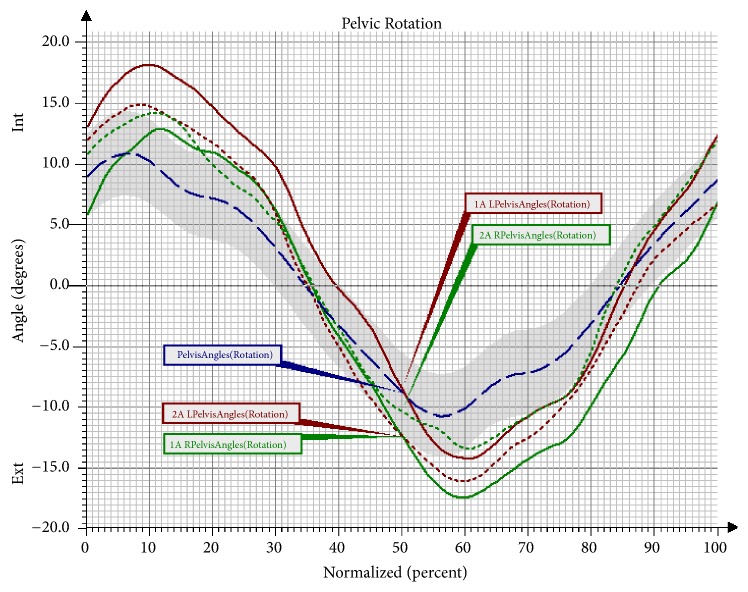
Angular changes in the movement of the pelvis in the transverse plane in Group NW before and after the training. Pelvis Angles: mean angular changes in the reference group; 1A Pelvis Angles: mean angular changes in the pelvis before the therapy; 2A Pelvis Angles: mean angular changes in the pelvis after the therapy; Int: internal rotation; Ext: external rotation; L: left; R: right.

After the applied therapy, the synchronisation of the movement of the pelvis improved; however, the observed changes were not statistically significant (Table 8; Figure 6).

**Figure 7:**
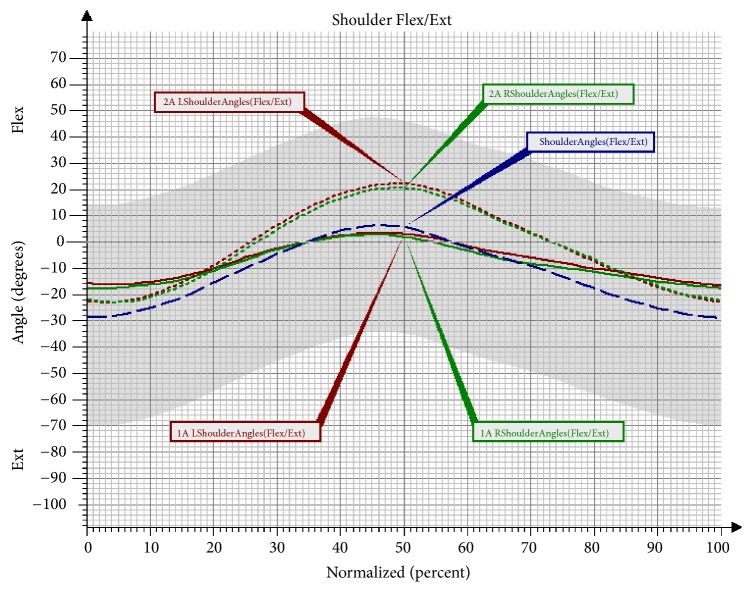
Angular changes in the shoulder joint in the sagittal plane in Group NW before and after the training. Shoulder Angles: mean angular changes in the reference group; 1A Shoulder Angles: mean angular changes in the shoulder joint before the therapy; 2A Shoulder Angles: mean angular changes in the shoulder joint after the therapy; Flex: flexion; Ext: extension; L: left; R: right.

The comparison of changes in the shoulder joint revealed a statistically significant increase of the flexion value in both upper limbs. A significant difference was also observed in the maximal extension value for the left upper limb (Table 9; Figure 7).

## 4. Discussion

The present study is the first one to discuss the topic of the effect of NW training in persons with Down Syndrome, in terms of the spatiotemporal parameters and angular changes in the joints while walking. Other studies available in the literature have focused on the effects of various other forms of physical activity. Moreover, to date no other studies have evaluated the angular changes during walking in persons with DS taking place simultaneously in the joints of the upper and the lower limbs.

We found that 10-week NW training programme induced changes in the gait parameters and improved the quality of walking in the adult persons with Down Syndrome. The obtained data allowed us to analyze the selected values of changes in the movement in selected joints of the lower and the upper limbs and to evaluate the movement of the pelvis in the studied persons. Furthermore, the analysed spatiotemporal parameters of gait allowed for a detailed assessment of the locomotion of persons with DS before and after the physical therapy. The comparison of the results between the two assessments conducted in the experimental group and the control group revealed statistically significant changes (p < 0.05) in a considerable number of parameters, taking place in the group that had trained in NW. After the 10-week training programme, the length of the step increased in both of the lower limbs, as did the length of the gait cycle. Regular NW training also significantly helped to increase the gait speed of persons with Down Syndrome.

Characteristic disorders of the gait in persons with DS concern both values of the spatiotemporal parameters and angular changes in the joints of the limbs. For the spatiotemporal parameters, interesting observations include a decrease of the gait speed and the related parameters, such as the cadence and the length of the step and gait cycle when compared to the model of a physiological gait [3]. The gait of persons with DS deviates considerably from the physiological standard for healthy persons, even from early childhood. A study conducted by Jung [20] involved assessing and comparing the gait of children with Down Syndrome and healthy children using the GAITRite System device. The results revealed that persons with DS already show significant locomotion disorders in childhood. Their gait is characterised by shorter steps, with a wider base of support and a lower speed of movement when compared to their healthy peers. What is more, the gait of healthy children improves with their development, while, in children with DS, it remains at a low level [20]. Evaluations of the spatiotemporal parameters of gait in persons with DS have also shown significant differences in young adults (16–22 years) when compared to the physiological gait. A study conducted with the Vicon 250 system revealed that the gait of persons with DS is characterised by different angular changes in the lower limbs and pelvis. The largest differences were observed in the range of the plantar flexion of the foot in the Terminal Stance phase and the Pre-Swing phase. In the studied group of persons with DS, the mean values of plantar flexion were 20° lower in comparison to those of healthy persons. Additionally, the studied persons maintained dorsiflexion at a mean value of 6° in these two phases of the gait. As was observed by Marchewka et al. [21], this fact may indicate that the triceps surae muscle is functioning ineffectively. A verification in the subject literature concerning the effectiveness of using NW training for gait re-education in persons with DS is difficult due to the scarcity of literature on this topic. There are, however, studies that have been conducted in other groups which indicate the effectiveness of NW training in improving the gait. Hagner-Derengowska et al. [22] compared the effect of an eight-week NW training programme and training that involved ordinary walking, which was applied in a group of healthy postmenopausal women. The groups that underwent the NW training obtained better biomechanical parameters of gait. Similarly, as in the present research, a significant increase of the gait speed and an increase in the length of the step were observed. Furthermore, the double support phase in the NW group became shorter, which again matches the trend observed in the present study. Another research hypothesis formulated in this study concerned the use of brisk NW walking to improve the peak angular values in the gait cycle. The observed significant changes lead us to the conclusion that the majority of angular values showed a positive trend after the NW training. The conducted NW training, in principle, affects the formation of the mechanisms of coordination and automatisation of the gait. It enforces longer steps, but it also activates the upper limbs, ensuring an increased mobility in the shoulder joints. As a result, it allows for a reinforcement of positive changes in the locomotion manner. The persons with DS, in the second assessment, showed a significant growth of plantar flexion in the ankle joint in the Loading Response phase for the right lower limb. This trend also occurred in the left limb; however, in this case the change was insignificant. The graphs created for the ankle joint function in Group NW indicate that the synchronisation of movement in the right and the left limb also improved.

Another observed change was a significant increase of flexion in the left knee joint in the Mid-Stance phase, as well as in the Terminal Stance phase and the Initial Swing phase in the group undergoing NW training. The increase in the range of movement in the knee joint is a desirable change that brings about many favourable consequences. The changes that took place in the hip joint after the NW training were not statistically significant; however, it should be observed that the curve of the angular changes for the right lower limb clearly approached the reference values. In the study there was also a trend observed involving an increased movement towards extension in the hip joint in the Pre-Swing phase, and, consequently, the effect of an increased synchronisation of movement in both lower limbs was obtained. The observed significant difference in the functioning of the pelvis in the sagittal and the frontal plane in the group training in NW should also be mentioned. The functioning of the pelvis, manifested through a decrease of the anterior pelvic tilt in the Mid-Stance phase and the Initial Swing phase, improved significantly. Furthermore, the NW training led to a decrease in the raising of the pelvis in the frontal plane and the falling of the pelvis. Few studies on gait have analysed the movement of the upper limbs. However, there is evidence that alternating swings of the upper limbs are a significant element in human locomotion. It has been proven that walking without an alternating movement of the upper limbs increases the metabolic cost of the walking, while walking with movement of the upper limbs reduces the energy cost by 8% [23, 24]. What is more, according to Meyns et al. [24], activating the upper limbs towards a swing may be a valuable therapy tool. So far, there have only been a few studies to discuss the role of the upper limbs in gait rehabilitation. However, it has been observed that excessive activation of the upper limbs in the studied persons can help to improve the gait speed and stepping frequency [25]. The effectiveness of the 10-week training programme in improving the work of the upper limbs during the gait cycle has been confirmed by the present study. An important result of the conducted research was an improvement in the mobility of the upper limbs during walking. A significant increase of flexion in the shoulder joints of both upper limbs was observed in the group that underwent the NW training. These changes were not observed in the control group, which indicates the effectiveness of the conducted therapy in activating the upper limbs during gait and eliminates the possibility that this improvement was caused by different factors.

It is also worth adding that the 10-week period of the experiment did not statistically affect the changes in the peak angular values for the group in which the therapy was not conducted. Nonetheless, the fact that the spatiotemporal parameters and the angular changes assessed during the normalised gait cycle did not change for the worse allows the conclusion to be made that, for both positive and negative changes to occur in gait, a longer period of time or an intensive, focused therapy is necessary.

As a limitation of our study, it should be noted that we have not analysed cognitive dysfunction and behavioural factors, which may confound evaluation of applied therapy. Moreover, we have enrolled 22 subjects to our study. From among them 11 participants took part in 10-week NW training. In future we aim to expand the research to larger study population and apply a longer period of therapy.

## 5. Conclusions

(1) The NW training improves the spatiotemporal parameters in persons with DS, causing elongation of step length and increasing the gait speed. Moreover, the NW training has beneficial influence on angular changes in the joints of the limbs, by approximating their average values to the physiological gait pattern.

(2) The NW training contributes to improvement of the mean values of angular deviations by increasing the joint mobility of the upper limbs during walking in persons with DS.

(3) Practical Implication: Regular NW training has beneficial impact in the field of rehabilitation and gait re-education in persons with DS; therefore it is recommended to include the NW training in daily activity.

## Figures and Tables

**Table 1:** Spatiotemporal parameters in Group NW and Group C before and after the training.

Parameter	Limb	Group	Before $\overset{-}{x}$ ± SD	After $\overset{-}{x}$ ± SD	F	P (time*∗*group)
Step length [m]	R	NW	0.46 ± 0.05	0.52 ± 0.05	14.47	**0.002**
		C	0.49 ± 0.1	0.48 ± 0.08		
	L	NW	0.48 ±0.07	0.53 ±0.04	5.15	**0.038**
		C	0.47 ± 0.1	0.47 ± 0.03		

Cycle length [m]	R	NW	0.91 ± 0.11	1.05 ± 0.09	14.48	**0.002**
		C	0.98 ± 0.19	0.97 ± 0.22		
	L	NW	0.93 ± 0.12	1.05 ± 0.06	15.09	**0.001**
		C	0.96 ± 0.21	0.95 ± 0.18		

Speed [m/s]	R	NW	0.76 ±0.12	0.87 ±0.15	8.51	**0.011**
		C	0.91 ± 0.27	0.9 ± 0.53		
	L	NW	0.76 ± 0.13	0.87± 0.14	8.22	0.12
		C	0.9 ± 0.28	0.9 ± 0.78		

L: left limb; R:right limb; NW: Nordic Walking group; C: controls; $\overset{-}{x}RL$: mean for the right and the left limb; SD: standard deviation; F: F value.

**Table 2:** Standardised (STD) spatiotemporal parameters in Group NW and Group C before and after the training.

Parameter	Limb	Group	Before $\overset{-}{x}$ ± SD	After $\overset{-}{x}$ ± SD	F	P (time*∗* group)
STD step length	R	NW	0.58 ± 0.08	0.66 ± 0.07	11.17	**0.004**
		C	0.63 ± 0.13	0.63 ± 0.13		
	L	NW	0.60 ± 0.10	0.66 ± 0.06	5.18	**0.038**
		C	0.61 ± 0.15	0.61 ± 0.15		

STD speed	R	NW	0.28 ± 0.05	0.31 ± 0.06	4.06	0.062
		C	0.33 ± 0.1	0.32 ± 0.11		
	L	NW	0.28 ± 0.05	0.31 ± 0.05	5.74	**0.030**
		C	0.32 ± 0.11	0.30 ± 0.14		
	$\overset{-}{x}R$L	NW	0.28 ± 0.05	0.31 ± 0.05	5.35	**0.035**
		C	0.33 ± 0.15	0.31 ± 0.17		

L: left limb; R: right limb; NW: Nordic Walking group; C: controls; $\overset{-}{x}RL$: mean for the right and the left limb; SD: standard deviation; F: F value.

**Table 3:** Mean peak values of angular changes in the ankle joint in Group NW before and after the training.

Gait phase	Limb	Angle of movement [°]		p
		Before	After	
LR	R	10.80 ± 14.49	-0.53 ± 4.85	**0.044**
	L	-4.28 ± 7.91	-0.97 ± 6.30	0.382

TS	R	25.69 ± 12.89	19.58 ± 5.56	0.184
	L	14.21 ± 4.32	18.98 ± 5.47	0.089

ISw	R	15.21 ± 10.99	6.39 ± 4.81	0.082
	L	-0.43 ± 5.89	1.86 ± 8.97	0.356

MSw	R	22.01 ± 11.76	16.90 ± 4.07	0.264
	L	9.11 ± 5.07	11.81 ± 4.26	0.169

LR: Loading Response; TS: Terminal Stance; ISw: Initial Swing; MSw: Mid-Swing; L: left; R: right; p: p value.

**Table 4:** Mean peak values of the angular changes in the knee joint in Group NW before and after the training.

Gait phase	Limb	Angle of movement [°]		p
		Before	After	
MS	R	28.39 ± 4.16	30.30 ± 4.57	0.221
	L	23.36 ± 6.05	28.25 ± 4.93	**0.002**

TS	R	17.62 ± 7.16	20.46 ± 5.08	0.066
	L	14.02 ± 6.83	18.22 ± 5.26	**0.017**

ISw	R	62.79 ± 5.83	65.90 ± 6.27	0.128
	L	60.26 ± 8.21	65.50 ± 5.82	**0.004**

MS: Mid-Stance; TS: Terminal Stance; ISw: Initial Swing; L: left; R: right; p: p value.

**Table 5:** Mean peak values of the angular changes in the hip joint in Group NW before and after the training.

Gait Phase	Limb	Angle of movement [°]		p
		Before	After	
IC	R	43.89 ± 5.11	42.90 ± 6.02	0.649
	L	40.96 ± 6.69	41.76 ± 6.17	0.755

PSw	R	10.21 ± 8.57	5.90 ± 6.57	0.054
	L	7.91 ± 8.56	5.21 ± 6.59	0.165

TSw	R	45.74 ± 5.98	44.97 ± 5.94	0.738
	L	44.31 ± 6.11	44.59 ± 7.13	0.896

IC: Initial Contact; PSw: Pre-Swing; TSw: Terminal Swing; L: left; R: right; p: p value.

**Table 6:** Mean peak value in the angular deviations of the movement of the pelvis in the sagittal plane in Group NW before and after the training.

Gait phase	Limb	Angle of movement [°]		p
		Before	After	
MS	R	18.43 ± 5.94	14.43 ± 5.14	**0.038**
	L	17.87 ± 6.13	15.22 ± 5.36	0.219

TS	R	20.21 ± 5.50	16.89 ± 4.81	0.106
	L	20.28 ± 5.89	17.62 ± 4.25	0.143

ISw	R	17.80 ± 6.09	15.24 ± 6.38	0.218
	L	18.63 ± 6.40	14.50 ± 4.62	**0.043**

MS: Mid-Stance; TS: Terminal Stance; ISw: Initial Swing; L: left; R: right; p: p value.

**Table 7:** Mean peak values in the angular deviations of the movement of the pelvis in the frontal plane in Group NW before and after the training.

Point	Limb	Angle of movement [°]		p
		Before	After	
Max	R	6.58 ± 3.03	6.38 ± 2.63	0.862
	L	7.50 ± 2.07	5.29 ± 2.18	**0.027**

Min	R	-7.67 ± 1.94	-5.21 ± 2.58	**0.002**
	L	-6.63 ± 2.84	-6.52 ± 2.55	0.921

A	R	14.24 ± 4.72	11.59 ± 4.21	0.076
	L	14.13 ± 4.71	11.81 ± 3.68	0.137

A: amplitude of values; p: p value.

**Table 8:** Mean peak values in the angular deviations of the movement of the pelvis in the transverse plane in Group NW before and after the training.

Point	Limb	Angle of movement [°]		p
		Before	After	
Max	R	14.55 ± 6.34	16.10 ± 3.73	0.449
	L	17.43 ± 5.06	17.11 ± 5.90	0.840

Min	R	-17.13 ± 5.28	-16.75 ± 5.76	0.840
	L	-15.11 ± 5.78	-16.69 ± 3.15	0.281

A	R	31.68 ± 9.05	32.84 ± 7.91	0.607
	L	32.54 ± 8.82	33.80 ± 6.78	0.633

A: amplitude of values; L: left; R: right; p: value.

**Table 9:** Mean peak values of the angular deviations in the shoulder joint in Group NW before and after the training.

Parameter	Limb	Angle of movement [°]		p
		Before	After	
Initial position	R	-17.76 ± 5.34	-21.27 ± 13.28	0.445
	L	-16.08 ± 6.92	-21.90 ± 12.51	0.173

Max	R	3.97 ± 11.93	18.69 ± 18.10	**0.021**
	L	4.96 ± 15.91	20.51 ± 16.66	**0.015**

Min	R	-16.31 ± 4.05	-20.97 ± 12.34	0.214
	L	-14.85 ± 6.84	-22.67 ± 9.43	**0.038**

L: left; R: right; p: p value.

## Data Availability

The datasets used and analysed during the current study are available from the corresponding author on reasonable request.
